# Extended seed rain period of *Adenostoma fasciculatum* impacts diverse seed predators

**DOI:** 10.1371/journal.pone.0250290

**Published:** 2021-06-15

**Authors:** Joanna M. Garaventa, V. Thomas Parker

**Affiliations:** 1 Department of Biology, San Francisco State University, San Francisco, CA, United States of America; 2 Garaventa Consulting and Research, Concord, CA, United States of America; Brigham Young University, UNITED STATES

## Abstract

**Aims:**

The principal chaparral species in California, *Adenostoma fasciculatum*, an evergreen, sclerophyllous shrub, is broadly distributed and provides habitat and food resources for a large and diverse animal community. The effects of climate change, including elevated temperatures, fire frequency and severity, along with increased urban encroachment, have placed pressure on chaparral habitats in California. Our goal is to investigate aspects of reproductive ecology as a measure of the potential resiliency of *A*. *fasciculatum*. We focus on seed rain (all seed falling into the seed traps regardless of origin) and seed banks in the context of plant-animal interactions and regeneration.

**Methods:**

Stand recovery following disturbance is achieved through both resprouting and germination from established persistent soil seed banks. In this study we focus on seed ecology using a series of experiments to document the length and quantity of seed rain, seed predation, parsing the importance of the community of granivores, and evaluating the connection between stand age and germination rate from soil seed banks.

**Important findings:**

Our research documented an 8-month seed rain duration with over 1 million seeds per m^2^, multiple seed predators including passerines (songbirds) and rodents, and points to the possibility of native ants playing a role in the seed dispersal process. This is important given the recent advancement of the invasive Argentine ant (*Linepthema humile*) into Californian chaparral. This research demonstrates a clear relationship between *A*. *fasciculatum* and both resident and migratory granivores in the chaparral. We documented that a 39-year-old stand had higher germination rates than those which were 16, 20, 41 and 71 years old and how seed banks play a major role in assuring resiliency following fire. These findings are important for wildland managers to assure the continued resiliency of *A*. *fasciculatum*.

## Introduction

For plants to persist in particular habitats, they must be able to survive the spatial and temporal variation in habitat stresses. Reducing risk at different stages of plant life histories is a critical product of natural selection interacting with the environment of the plant [1]. California chaparral is a semi-arid vegetation that provides multiple biotic and abiotic constraints, dominant plants of which may exhibit variation among life history stages. Covering a broad geographic expanse and climates within California (approximately 23% of land area), chaparral includes nearly a quarter of the plant diversity of the California Floristic Province [2, 3]. The Mediterranean-climate of California chaparral hosts multiple stresses for plants, from the characteristic summer rainless periods and subsequent wildfires to variation in geology and animal communities of mutualists and antagonists. Multiple life history combinations of plants in this vegetation illustrate multiple solutions to these stresses. Among the woody dominants are three ways plants cope with drought and fire, from deep-rooted, obligate sprouters; relatively deep-rooted facultative sprouters, and shallow-rooted obligate seeders. These coarse classifications omit critical details, for example, seed dispersal and patterns of germination varies greatly among species [4] and even among the three principal shrub genera with persistent seed banks their dispersal and dormancy breaking processes differ in combinations. *Ceanothus* (Rhamnaceae) species, for example, produce explosive fruit and the seed suffer intense seed predation yielding low density seed banks [5, 6]. *Arctostaphylos* (Ericaceae) species mature dry drupes that are mammal dispersed with scatter-hoarding rodents caching them at depths sufficient to survive fire [7]. *Adenostoma fasciculatum* (Rosaceae), the principal dominant of chaparral throughout the region at lower elevations, is less studied for these traits. Its’ small dry achenes appear to be passively dispersed, yet the species seems to be able to produce dense seed banks [8]. We investigate seed production, dispersal, seed predation, and seed bank formation with the objective of understanding these stages in more detail.

*Adenostoma fasciculatum* evolved during the Paleogene (43–66 MYA) [9]; it is now tolerant of both summer dry periods and canopy wildfires. The onset of the Mediterranean-type climate system (summer dry/winter rain) may have been during the Miocene and the evolutionary adaptation of *A*. *fasciculatum* to a summer-dry and winter-rain climate with high-intensity seasonal fires has yielded a complex regeneration strategy which includes the ability to regenerate via stump sprouting from lignotubers [10–13] and seed germination via a polymorphic seed bank [8, 14]. This dual strategy is critical as *A*. *fasciculatum* exhibits relatively high mortality of burned adults in either high intensity fires or subsequent droughts [15, 16]. The seed bank consists of two types of seeds, the first are transient in nature, requiring no specific cue for germination, and the second are dormant and germinate in response to fire stimulation [10, 13, 17]. As a critical method of regeneration, seed banks of *A*. *fasciculatum* act as a biological reservoir ensuring regeneration after a fire and permit persistence with unpredictable environments [18].

The abundant seed production of *A*. *fasciculatum* also suggests the potential for a variety of interactions with diverse animal granivores both resident and migratory. Stands of *A*. *fasciculatum* produce a dense low growing canopy which creates optimal habitat for many animals, particularly rodents and passerine birds (songbirds) [7]. Rodents frequently utilize the branches of shrubs as pathways throughout the stand thus avoiding ground-dwelling predators. Many species create dens within the soil beneath these shrubs. Similarly, birds can nest within the dense canopy of *A*. *fasciculatum* thus avoiding some predators while having a ready food source nearby. Many of these animal species are principally granivores. It is unclear the extent to which seed predation occurs in *Adenostoma* stands, but it appears to be much less than with *Ceanothus* or *Arctostaphylos* species [6, 8, 19, 20].

How *Adenostoma* disperses is not yet clear. These dry achenes exhibit no adaptations for wind dispersal [4]. Currently no published quantitative data exists on the duration of seed rain in *A*. *fasciculatum* stands as well as the timing of dispersal, nor how these patterns may relate to potential chaparral granivores, their behaviors, and phenology. The relationship between some chaparral plants and granivores through secondary dispersal (diplochory) has been well documented and previous chaparral studies have recorded seed preferences of rodents and their role as secondary dispersers through scatter hoarding and seed caches [21]. Several studies have documented ant-mediated seed dispersal in grasslands and other Mediterranean habitats [22–25] through caching of seeds within or near nests. This appears to be particularly significant in semi-arid habitats [4]. Whether ants play a role in *A*. *fasciculatum* seed dispersal is unknown. The behavior of many ant species to clear areas surrounding their nests may lead to soil seed bank deposition. Depending on the depth of deposition, such caches will contribute to germination.

*Adenostoma fasciculatum* stands are generally long-lived; documented stand age based on fire intervals has exceeded 80 years [26–29]. As a stand ages, the overall biomass of the stand eventually decreases over time and seed deposition rates decline [16]. Seed banks of *A*. *fasciculatum* are maintained with a significant seed rain; one study determined seed bank densities ranging from 2000 to 21,000 seeds per m^2^ depending on the age of the stand [8]. Its’ seeds can germinate lower in the soil profile than those of desert or grassland seeds–more than 5 cm depth–thus ensuring that some seeds remain protected from high soil temperatures generated during fires [4, 8, 30]. Consequently, post-fire seedling densities can be considerable in the first year [14, 31, 32]. Post-fire germination and seedling densities seem to fall into two patterns. In some cases, older stands have higher germination rates than younger ones simply due to a larger soil seed bank [8, 33–36]. In contrast, the most productive age for seed germination may lie somewhere between 30 and 40 years of age, declining thereafter [8]; for example, germination rates of younger and older stands show far lower germination rates than mid-aged stands (~30–40 yrs.) [8, 34, 35]. Mid-range maximum germination rates occur in other plant species (*Genista monspessulana*) [36]. If mid-aged stands have higher overall germination rates, it may be due to their continued ability to deposit large amounts of seeds to the seed bank despite granivory and loss of viability in older seed cohorts. Because the soil seedbank is continually fluctuating with new seeds being deposited balanced against seed consumption and viability loss in older seeds, declining seed production with age would result in lower post-fire germination rates in older stands. soil seed bank [7, 10].

*Adenostoma fasciculatum* has a complex life history that undoubtedly underlies its dominance in chaparral throughout the California Floristic Province. Changing climate in California indicates increased temperatures and a longer rainless period, potentially modifying chaparral fire regimes by increasing fire frequency and severity [37, 38]. Anthropomorphic manipulation of chaparral has led to attempts for restoration at some sites [33, 39]. While life history data on *Adenostoma* is sufficient to provide some estimate of these impacts on recovery after fire, much less is known about its animal interactions and their resiliency to change, especially in the context of evolving fire regimes. Our overall objectives are to test some of the past patterns seen with respect to seed production and seed banks, while expanding on other less studied dimensions. Theoretical and empirical studies already have indicated that there can be various trait dimensions to persistence of a plant population in a habitat [40–43]. That has led to studies investigating the extent to which population persistence may reflect trade-offs between, for example, dispersal and seed dormancy, depending on the predictability of successful seed germination and seedling establishment [44–46]. These trade-offs have been called bet hedging, and while bet hedging at the seed bank stage is well acknowledged [35, 47, 48], other life history stages have also exhibited diverse responses to environmental stresses [49–51]. Recognition of their significance is reflected in a series of recent attempts to set up frameworks for dispersal, seed banks, and seed dormancy [52–55], for example, describe aspects of seed-trait functional ecology which incorporates categories of dispersal, persistence, germination timing and establishment. Patterns found in *Adenostoma* may expand on the predictions of these frameworks due to its multiple potential responses. Consequently, our specific study aims are fourfold: to clarify seed production and dispersal patterns by determining the rate, length, and peak seed deposition period of a mid-aged *A*. *fasciculatum* stand; to determine whether a correlation exits between stand age and seed bank seed germination across multiple stands; to document the community of animals which feed on *A*. *fasciculatum* seed using motion-activated wildlife cameras; and to determine whether there is a differential preference among insects, birds or rodents in seed consumption.

## Materials and methods

### Site selection

Five sites were selected with chaparral dominated by *A*. *fasciculatum* within the San Francisco Bay Area, California (U.S.A.). Sites were located within the inner and outer Coast Ranges: Del Valle Regional Park in Livermore, California, a 16 year old *A*. *fasciculatum* stand (37°34’16.69”N/121 41’ 43.20”W, elevation 382 m); Marin Municipal Water District, Marin County, California, with 2 *A*. *fasciculatum* stands, 30 and 71 year old sites (37°55’12.62” N/122 34’ 51.01”W, elevation 64 m); Mount George Foote Botanical Area Preserve in Napa, California, a 41 year old *A*. *fasciculatum* stand (38°17’10.11”N/122 11’31.17” W, elevation 334 m); and Mt. Diablo State Park, Contra Costa County, California, a 39 year old *A*. *fascisculatum* stand (37°51’54.92”N/121 55’ 36.09” W, elevation 829 m). These sites were selected as they represented stands with a broad age range (16 to 70 years) and were all dominated by *A*. *fasciculatum*. The Mt. Diablo stand was used for most experiments.

Field site access for all sites was secured prior to sampling being conducted by permits or permission issued by the following staff and entities: East Bay Regional Park District (EBRPD), Del Valle Regional Park, Livermore: EBRPD Supervisor Denise Defreese; Marin Municipal Water District watershed; Janet Klein, Natural Resources Program Manager; Mount George Foote Botanical Area Preserve, Napa, California, Mike Palladini, Stewardship Program Manager, Land Trust of Napa County; Mount Diablo State Park, Contra Costa County, California; Christina Freeman, Park Supervisor.

### Seed rain

**Fig 1:**
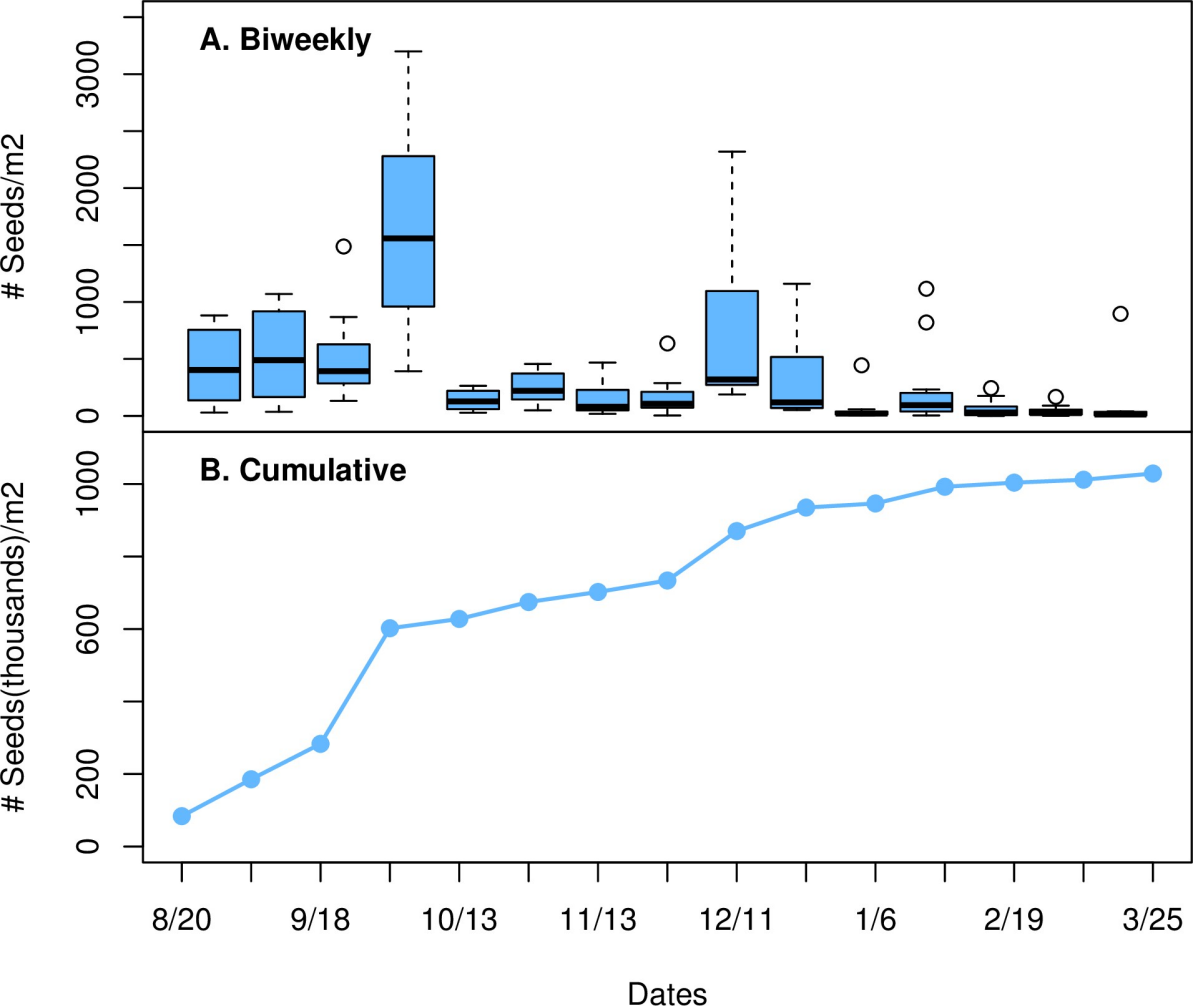
Seed rain. a) Mean Bi-weekly *A*. *fasciculatum* seed rain per m^2^ from the mid-aged, 39 yr. old site (Mt. Diablo), August 2016—March 2017; b) cumulative seed rain totals for the same time period.

Annual seed rain was monitored on a bi-weekly basis within a single site at the Mt. Diablo location on a south-facing slope at 829 m elevation from August 2016 to March 2017 (Fig 1). The annual rainfall during this time was 675 mm with average annual rainfall being 515 mm per year. Twelve individual seed rain stations were selected along an approximately 250 m transect paralleling the slope using a nested sample design. Individual station locations were determined using a random number table. Each collecting station contained two 25 x 25 cm trays (0.0625 m^2^): one was covered with hardware cloth (0.69 cm openings) and the other uncovered. Each tray was secured to the ground using metal U-shaped landscape staples to prevent movement and predation of seeds within the covered trays.

Seeds from each of the twelve covered trays were collected, bagged, labeled, and brought back to the lab on a bi-weekly basis. Seeds within the uncovered trays remained in place until the end of the study in March 2017 at which time they were bagged, labeled, and transported back to the lab for counting. Seed rain samples were sieved to eliminate detritus and then counted. Only whole seeds and those which were less than 50% consumed were included in the count.

### Seed predation by granivores

Motion-activated wildlife cameras were placed at six of the twelve seed rain collection stations within the Mount Diablo study site. Data from motion-activated wildlife cameras were collected from SIM (Subscriber Identity Module) cards and analyzed on a bi-weekly basis. Video data was reviewed to determine frequency of visits by type of species.

### Differential predation testing

Four additional seed station locations were established within the same *A*. *fasciculatum* stand at the Mount Diablo site at the culmination of the seed rain study. Each seed station location contained three 0.0160 m^2^ (20 x 8 cm) area containers providing varying degrees of access for granivores to seed: one uncovered station to allow full access to seeds by either rodents or birds; the second which was partially covered by hardware cloth screen (0.69 x 0.69 cm openings) elevated approximately 2.5–3.0 cm above the station to exclude birds but permit access by rodents and insects; and, the third station completely covered by the hardware cloth screen to permit access to only insects.

Motion-activated wildlife cameras were set-up at each of the four sites and a total of 100 seeds were added to each container. Seed stations were checked each week for two weeks to assure seed offering was still adequate. Video data from SIM cards were collected from cameras each week.

### Seed bank germination rate by stand age

Twelve soil seed bank samples were collected from the five separate sites for estimating seed bank sizes. Each soil seed bank sample was collected in two parts: the upper 2 cm and the lower 3 cm collected separately. The age of each stand was determined using documentation supplied by park management and CalFire data except for the Del Valle site. The age of this stand was determined by cutting 3 primary branch sections from individual random obligate seeding shrubs within the stand, then cutting a 2.0 cm thick section from each, counting growth rings under a dissection microscope, and averaging the results.

Seedbank samples were collected at random points along a transect bisecting each site using a random number table. A 0.0625 m^2^ area quadrat frame (25 x 25 cm) was utilized for collection of the upper 5 cm of soil. Soil seed bank samples were collected in a stratified manner with the upper 2 cm of soil being collected and bagged separately from the lower 2–5 cm of soil. Samples were labeled and transported back to the lab following collection. A total of 120 separate samples were collected.

Preparation of each sample included: air drying in an open container and sieving through a 0.69 cm mesh to eliminate large rocks, twigs, and other non-soil debris. Samples were dried in a 71°C oven for 30 minutes to mimic a fire heat pulse, cooled, and transported to the greenhouse, and spread on 2 cm of clean sand in individual 25 x 25 cm trays. Samples were watered weekly with a dilute mixture of pyroligneous acid (~1:1000) and gibberellic acid (0.01 ppm) to facilitate germination and monitored to assure adequate moisture. After several weeks, samples were transported from the greenhouse to a netted outdoor area to provide natural ambient temperatures.

Potential differences among sites and between layers was tested by ANOVA. Linear and polynomial regression models were used to fit to the seed bank data.

### Analyses used in study

#### Seed rain

Cumulative seed rain count over test period.

Determination of peak seed rain period.

Determination of maximum bi-weekly seed rain.

Wilcoxon Sign-Rank test and paired t-test of data to determine significance between open vs. closed trays.

#### Seed predation by granivores

Analysis of motion-activated wildlife camera data to determine timing and frequency of visits to seed traps by granivores.

Compilation of a list of granivores visiting traps.

#### Differential predation testing

Analysis of differential between initial seed offering and seeds remaining at the end of the test period.

Analysis of motion-activated wildlife camera data to determine types of granivores visiting traps.

#### Seed bank germination rate by stand

Best-fit analysis using a linear-regression model.

## Results

### Seed rain

Seed rain occurred continuously from August 2016 through March 2017 (Fig 1A). Peak seed rain occurred from the end of September to mid-October 2016 with a second smaller peak noted in early December 2016. During the peak period, mean maximum bi-weekly seed rain was 44,288 seeds m^-2^. During the secondary peak period mean maximum bi-weekly seed rain was 28,416 seeds m^-2^. Cumulative seed rain over the duration of the study (August 2016 to March 2017) was calculated to be more than 1 million seeds m^-2^ (Fig 1B).

### Seed predation

**Fig 2:**
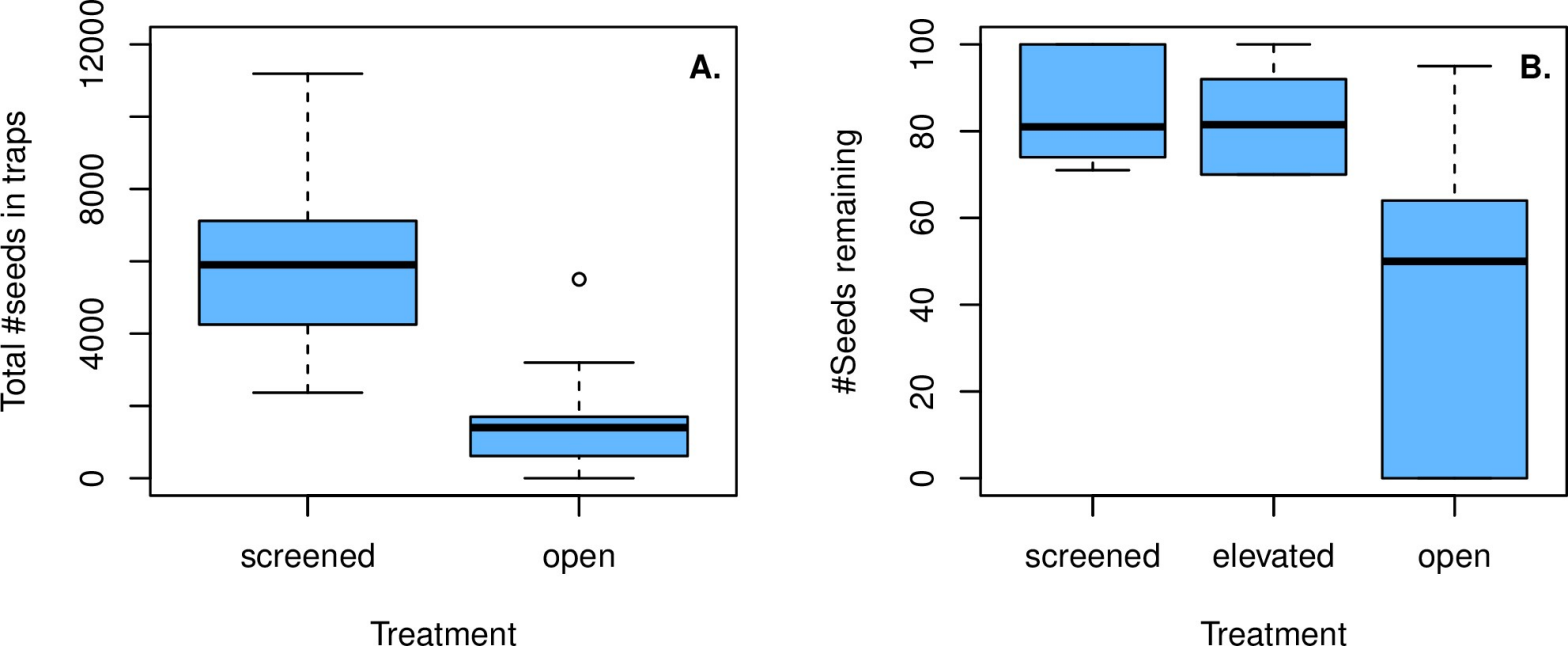
a) Average screened vs. open tray seed count at the Mt. Diablo site (paired t-test p = 3.413e-06; b) predation experiment using screened, elevated screens and open trays.

Seed predation in open trays was substantially higher than screened (closed) trays, with open trays having approximately 75% of their seeds consumed using closed trays as the baseline. A Wilcoxon Sign-rank test of open vs. closed mean seed tray counts yielded a P-value of 0.0004883 and a paired t-test p = 3.413e-06. Both tests showed a statistically significant difference between the two treatments of open vs. closed seed traps (Fig 2A and 2B).

Granivore species consisted primarily of rodents and passerines. *Peromyscus maniculatus* (Deer Mouse) and *Neotoma cinerea* (Bushy Tailed Woodrat) were the primary rodent granivores and *Catharus guttatus* (Hermit Thrush) and *Junco hyemalus* (Dark-eyed Junco) the primary passerine seed predators. Rodent predation seemed to follow the initial fall peak seed rain while passerine predation was highest during the winter and early spring months. Migratory species of the bird species *Zonotrichia atricapilla*, (Golden-Crowned Sparrow) and *Spinus psaltria*, (Western Goldfinch) were also found to browse on *A*. *fasciculatum* seed during the fall and winter months. This is of interest as it suggests that migratory bird species may utilize *A*. *fasciulatum* seed as a food source during their over wintering in California. Resident birds were also observed eating at the seed trays: *Melazone crisalis* (California Towhee), *Sitta carolinensus* (White Breasted Nuthatch), and *Pecile rufensens* (Chestnut-backed Chickadee) and the majority of observed predation was by resident passerines (Table 1).

**Table 1:** Number of visits by species to seed trays as captured by motion-activated wildlife cameras. Mt. Diablo State Park. August 2016 to March 2017.

			2016				2017				
		Aug	Sep	Oct	Nov	Dec	Jan	Feb	Mar	Apr	Total
*Catharus guttacus*	Hermit thrush			2		33	1	37		4	77
*Sitta carolinensus*	Nuthatch			1		1					2
*Spinus psaltria*	Goldfinch					3					3
*Junco hyemalus*	Dark Eye Junco					27	34	29			90
*Dipodomys californicus*	Kangaroo rat			10		1					11
*Passer domesticus*	English sparrow		1	2							3
*Prenolepis imparis and Veromessor sp*.	Smooth Harvester Ant or False Honey Pot Ant		3	1	3						7
*Peromyscus maniculatus*	Deer Mouse			17	3	30		8		1	59
*Neotoma cinerea*	Bushy tailed wood rat			8		9		9		4	30
*Pecile rufensens*	Chestnut-backed chickadee					1		1			2
*Melazone crisalis*	California Towhee									3	3
*Zonotrichia atricapilla*	Gold Crowned Sparrow						1	2			3
	**Total**	**0**	**4**	**41**	**6**	**105**	**36**	**86**	**0**	**12**	**290**

**Fig 3:**
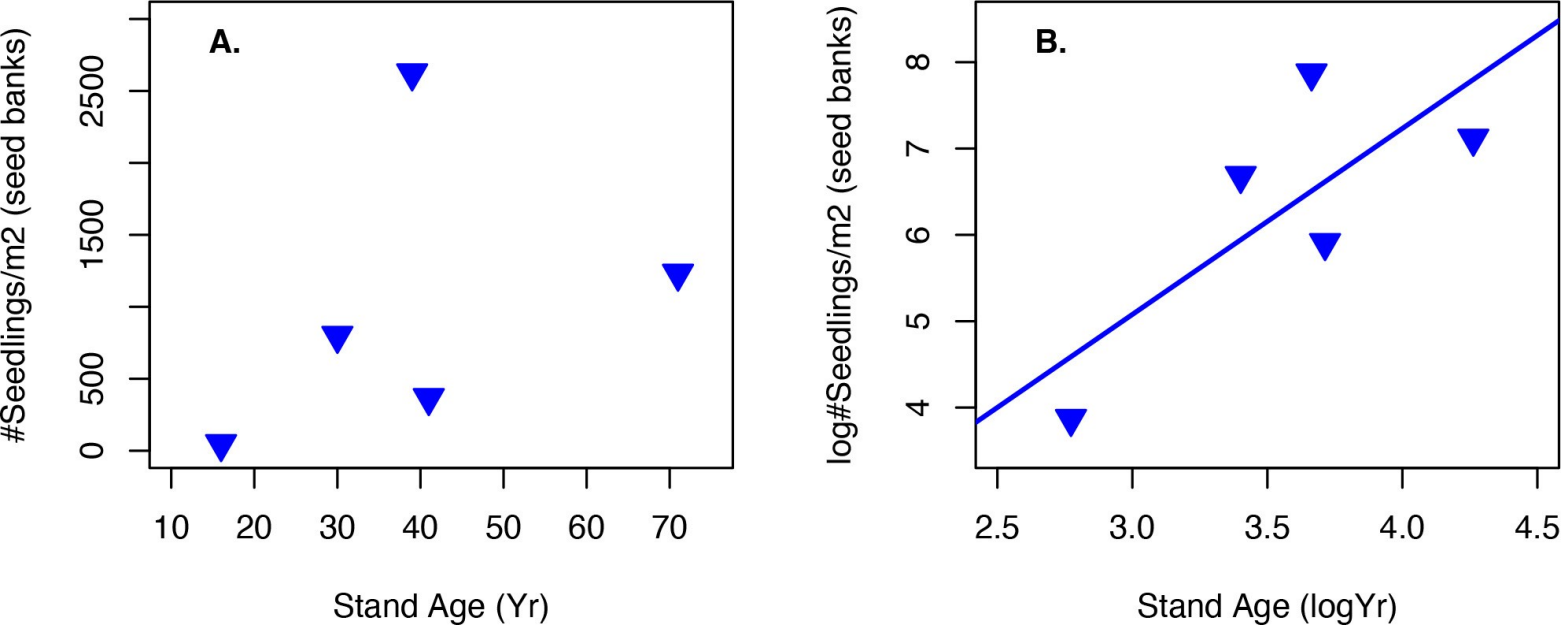
a) Seed bank germination totals at five test sites of different ages from the San Francisco Bay region, California; b) best fit model was a log-log model for the seed bank germination data.

Insect consumption of seeds was documented during two time periods: a) August to September 5–9% consumption; and b) October to November when 2–4% of all seeds showed signs of insect predation. Insect predation was not documented during the remainder of the study period. Insects found in seed rain samples included ants *Prenolepis imparis* (winter ant) and *Veromessor sp*. (Smooth harvester ant) and Dirt-colored seed bugs (*Rhyparochromidae)* (Fig 3A).

### Differential seed predation

In the differential seed predation experiment, the largest number of seeds consumed across all treatments during the three-week test period occurred in open trays with 57% of seeds removed (341 of the 600 seeds). Elevated screens and closed (screened) trays had approximately the same amount of total seed predation during the same period (105 and 93 seed lost, 17.5% and 15.5%, respectively). Paired t-tests were performed on the data with Open vs. Closed trays and Open vs. Elevated trays showing significant differences (p-values of 0.032 and 0.039 respectively). Elevated vs closed trays were not different (P = 0.43; Fig 2B). Motion-activated wildlife camera observations were made of rodents in both the open and partially covered trays and ants were observed only in the covered trays.

### Seed bank germination

Germination rate from soil seed bank collections by stand age was tested across five sites ranging in age from 16 to 71 years of age. Stand age was defined as time since last fire and germination rates by seedling emergence. Maximum germination rates occurred in soil from the 39-year-old stand, with the next highest germination rate by the 71-year-old stand. Germination rates in the lower 3 cm of soil were consistently higher in all five locations (F_9,110_ = 3.286, p = 0.0014). Seed density in the upper 2 cm was about half (52.5%) that of the lower 3 cm, thus representing only 34.4% of the seed bank for the top 5 cm of soil depth. While total germination visually supports the mid-stand age having peak densities, no linear or non-linear regression model was a statistically significant fit, possibly due to the small number of stands and their variance; the best fit was a log-log linear model although it was not significant (Table 2; Fig 3) (multiple R-squared: 0.5832, adjusted R-squared: 0.4442, F_1,3_ = 4.197, p-value = 0.1329).

**Table 2:** Linear model fits to seed bank germination data.

Regression model	AIC	BIC	F-statistic	p value
both seed # and age log transformed	18.93804	17.76635	4.197_1,3_	0.1329
seed # log transformed	20.79688	19.62519	1.962_1,3_	0.2558
age log transformed	59.24235	58.07067	0.8349_1,3_	0.4282
untransformed data	59.74693	58.57525	0.4668_1,3_	0.5435
fitting a polynomial	60.524535	58.96231	0.4756_2,2_	0.6777

## Discussion

### Seed rain

*Adenostoma fasciculatum* has been noted to have a summer seed dispersal period [2]. We noted a much more extensive seed dispersal period, in the range of 8-months, which in our study began in August and ended in March. To our knowledge, the lengthy seed rain of approximately 8 months duration was not previously documented for this species and is atypical of other chaparral shrubs which tend to have short bursts of seed rain which last only a few to multiple weeks [4]. Most taxa found with delayed dispersal like this are from desert plant communities [55–59], salt marshes [56], or Mediterranean-region shrub communities [60]. The lengthier seed rain suggests a risk-reducing response to extensive seed predation [50], providing sufficient seed for granivores, but extending dispersal past the activity periods of different types of seed predators, potentially allowing for some seed incorporation into the soil seed bank. Thus *A*. *fasciculatum* retains seed on its panicles as temporary aerial seed banks, incrementally depositing seeds from summer through early spring thus increasing the likelihood that seeds may bypass granivores and be preserved in the soil seed bank (Fig 3).

The long dispersal phase is paired with high seed production. While others have documented relatively high seed production values in *A*. *fasciculatum* [8], we know of no studies with numbers as high as we found. The large number of seeds dispersed (in our sample year over 1 million m^-2^) attracted a broad community of granivores, similar to other arid and semi-arid communities [58, 61, 62]. Variation in seed bank density and depth distribution patterns overall suggests the interpretation that granivory has a major impact on soil seed banks in this species [6, 62]. Finally, the type of granivore that dominated seed removal shifted during the long seed rain period of the stands.

### Seed predation

Some seeds within the seed rain samples were observed to have potential pre-dispersal seed predation. Seeds collected during the fall period were eaten by insects as they contained insect bore holes and frass. The duration of this phenomenon was short (11 weeks) and did not reoccur in later samples. While it is not known for certain which insects caused this damage, several members of the *Rhyparochromidae* family (dirt colored seed bugs) were found in the seed samples. These species of insects are known seed eaters and their presence in the seed traps suggests they were among those that utilize *A*. *fasciculatum* as a food source.

The animal community which utilizes *A*. *fasciculatum* seed following initial dispersal as a food source is quite diverse and includes several rodent, passerine, and insect species. The granivorous rodent and bird species are common in the region and known to be efficient seed predators in chaparral [6, 7]. In arid areas of North America, these three groups commonly occur together as seed predators on the same species, generally with rodents having a greater impact, and ants and birds varying in impacts by species [17, 18, 61, 63–65]. The temporal patterns of foraging among these animals may have selected for the lengthy seed dispersal period.

Although not mentioned in previous research, ants were frequently found in seed trays and in bi-weekly samples. The area surrounding the site is filled with ant hills of *Veromessor sp*. (Smooth Harvester Ant) and several specimens of *Prenolepsis imparis* (False Honey Pot Ant) were found in seed tray samples. Red harvester ants (*Pogonomyrmex barbatus*) were frequently seen in stands of *A*. *fasciculatum* used in this study that were relatively open. Possibly these ant species are collecting and storing *A*. *fasciculatum* seeds in their burrows as a winter food source since ants prefer small seeds which are easy to carry [23]. Ant caching of seeds has been shown to be beneficial to some plants as seeds are shallowly buried thus making them more likely to benefit from phytochrome stimulation, water imbibition and ultimately germinate [25, 60]. In the case of *A*. *fasciculatum*, ant dispersal could result in seeds being deposited into soil gaps below 5 cm which could result in germination following a fire. This process has been shown to be of significant benefit in grassland ecosystems as well as other Mediterranean habitats [22, 66]. Further research which documents the potential role of ants as an *A*. *fasciculatum* seed disperser would be beneficial. Ants have been documented to play a dispersal role in only two other chaparral endemics, *Dendromecon rigida* (Bush Poppy) and *Fremontodendron decumbens* (Pine Hill Flannelbush) [64, 65, 67]. Ants may play a similar role regarding *A*. *fasciculatum*.

The role of ants and seed bugs as seed predators and possible seed dispersal agents was more extensive than expected. Ants as a possible secondary disperser has not been explored for this species and is of interest given the recent advancement of invasive species such as the Argentine ant (*Linepithema humile*) into Californian chaparral and the potential for displacement of native ant species [68, 69]. *Linepithema humile* has been documented to have immigrated into California as far north as the San Francisco Bay Area. It displaces native ant species by occupying their nests and creating “super-colonies” which extend over a broad geographic area [68]. Being non-granivorous by nature, the influx of this species could significantly affect the role which native ants play in their relationship with *A*. *fasciculatum* through secondary dispersal and micro-caching. Without seed removal and caching by native ants, there could be increased opportunity for predation by other granivores thus decreasing the number of seeds that are annually deposited in the soil seed bank.

**Fig 4:**
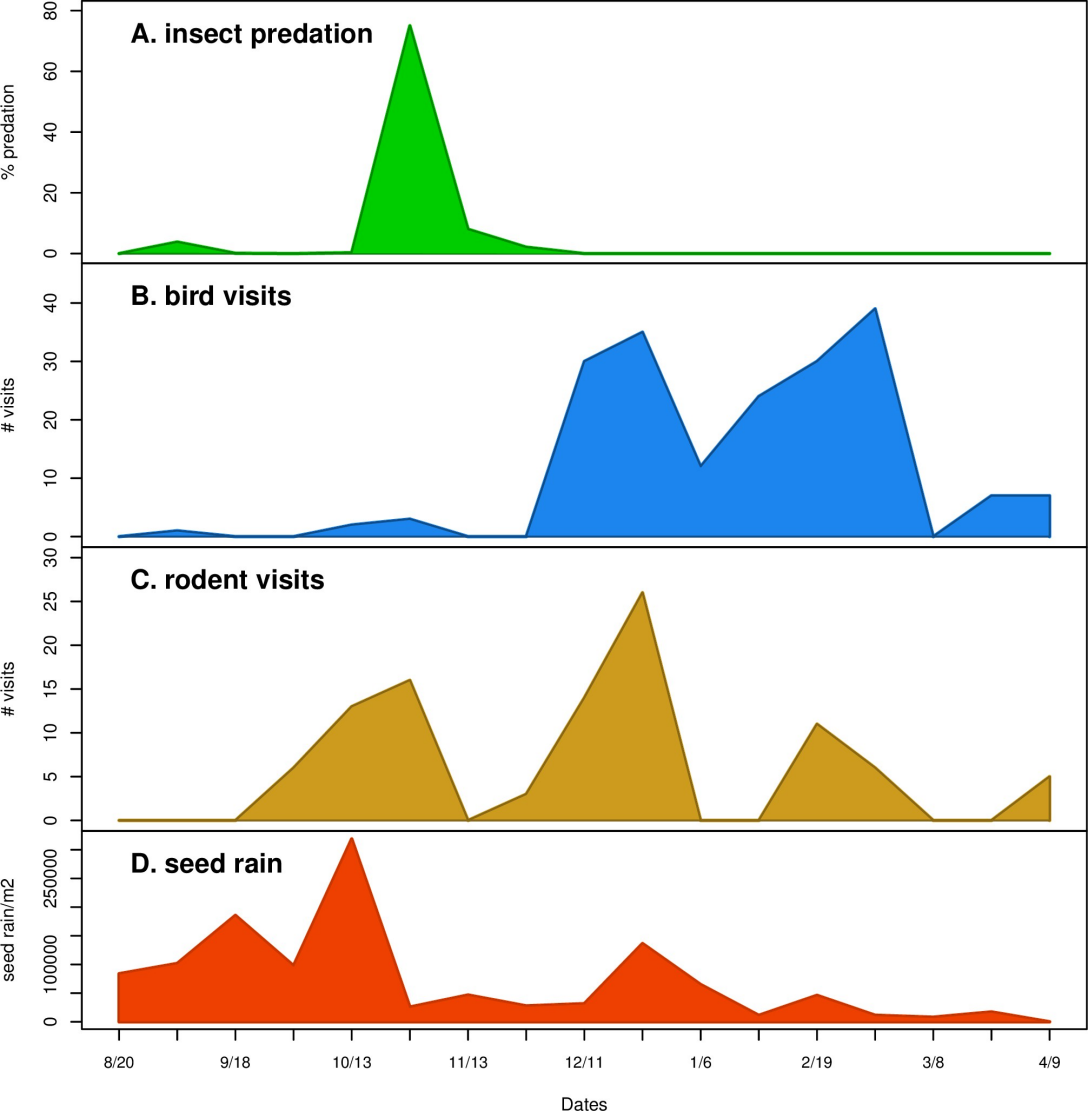
Temporal comparisons of a) insect; b) bird; and c) rodent visitations as captured by trail cameras or damage to seeds. These are in the context of d) the pattern of seed rain. Fires in this vegetation occur during the dry summer and overlap with the dispersal timing, typically peaking in September; while climate shifts have extended the fire season both earlier and later, the peak so far has remained September to early October.

Temporal patterns between seed dispersal and granivore-types indicate a sequential shift of dominance among types of granivores. Analysis of seed predation by species type (Fig 4) portrays a temporal relationship between seed rain and three groups of animal seed predators: insects, rodents, and passerines. Insect predation, while limited to a 3-month period, coincides with peak seed rain deposition [67]. Rodent predation closely follows peak seed rain deposition periods. Passerine predation in *A*. *fasciculatum* stands seems to be unrelated to seed rain peaks or duration as it occurs primarily during winter months and perhaps is associated with migratory patterns or the lack of food availability elsewhere.

The high seed production and the lengthy dispersal period suggests strong selection by granivory [68]. Correlation between substantial seed production years and subsequent reproductive output of rodents and birds has been validated by previous studies that demonstrate a positive long-term effect on reproductive success of small mammals and birds [68, 69]. Passerine predation may be connected to the need for increased food sources as nesting season approaches in early spring. In addition, migratory birds may utilize *A*. *fasciculatum* seeds as a food source during their overwintering in California or during their migratory passage south. Notably, *A*. *fasciculatum* stands generally follow the north-south passerine migratory trajectory along the California coast.

### Seed bank germination

The seed bank germination study corroborates previously held hypotheses that seed bank density is related to stand age as the middle-aged stand (39 years old) had significantly higher rates than either younger or older stands [4, 8, 29, 62]. The best fit model, however, was a linear model suggesting that seed banks may continue to increase in size through time and we just had too few sites to distinguish among hypotheses.

Of the 5 cm of soil collected for seed bank study, soil depth differed in seed density with the lower 3 cm of soil having significantly higher germination rates than the upper 2 cm in contrast to many species [69]. The dynamics of high predation rates at the surface and shallow depths also combined with non-refractory seeds not persisting, may explain the differential pattern of slow accumulation of seeds at depth. Few seedlings germinated within several of the five seedbank sites (16, 30 and 41 years since last fire). This may indicate younger stands have not had sufficient time to develop a substantial seed bank in the context of lower seed production and high seed predation rates [29, 35]; while older stands may exhibit declining rates of seed production and potentially loss of viability in older seed cohorts [29]. Soil seed density may be affected by multiple factors including the resident animal community, the type of soil, amount of annual precipitation, pathogen attack, and whether the seed contains enough reserves to maintain the seed embryo. Given only five sites were tested in this study it is difficult to extrapolate more generally.

*Adenostoma fasciculatum* is the dominant species throughout most of the range of chaparral but climatic shifts place this species particularly at risk [38]. Chamise stands provide habitat and food for a wide variety of resident and migratory animals. The prodigious production of seeds combined with the extended seed rain period undoubtedly have contributed to its broad distribution range throughout California chaparral. This extended seed rain period accommodates a diversity of granivores including insects, but more work is required to understand the full breadth of plant and animal mutualisms and interactions within this community. The documentation of significant plant-insect interactions suggests future work could also focus on pre-dispersal seed predation and documentation of insects which may utilize *A*. *fasciculatum* flowers and dispersed seeds.

## Supporting information

S1 Data (XLSX)
